# Updated Perspectives on Lifestyle Interventions as Secondary Stroke Prevention Measures: A Narrative Review

**DOI:** 10.3390/medicina60030504

**Published:** 2024-03-19

**Authors:** Valbona Govori, Hrvoje Budinčević, Sandra Morović, Filip Đerke, Vida Demarin

**Affiliations:** 1 Department of Neurology, University Clinical Center, 10000 Prishtina, Kosovo; 2 Department of Neurology, Sveti Duh University Hospital, 10000 Zagreb, Croatia; 3 Department of Neurology and Neurosurgery, Faculty of Medicine Osijek, J. J. Strossmayer University of Osijek, 31000 Osijek, Croatia; 4 Aviva Medical Center, 10000 Zagreb, Croatia; sandra.morovic@poliklinika-aviva.hr; 5 Department of Neurology, Dubrava University Hospital, 10000 Zagreb, Croatia; 6 International Institute for Brain Health, 10000 Zagreb, Croatia

**Keywords:** recurrent stroke, secondary prevention, lifestyle modification, health behavior, physical activity, air pollution

## Abstract

Despite being highly preventable, stroke is the second-most common cause of death and disability in the world. Secondary prevention is critical as the stroke recurrence risk is 6- to 15-fold higher than the risk of stroke in the general population. Stroke recurrence is associated with higher mortality rates and increased disability levels. Lifestyle modifications should address not single but multiple cardiovascular risk factors to effectively reduce the risk of stroke. Lifestyle modifications on a personal level should include adequate physical activity, a healthy diet, the cessation of smoking and alcohol consumption, and stress reduction. Physical activities should be performed in a healthy environment without air pollution. According to recent studies, up to 90% of strokes might be prevented by addressing and treating ten modifiable stroke risk factors, half of which are related to lifestyle modifications. These lifestyle modifications, which are behavioral interventions, could impact other modifiable risk factors such as arterial hypertension, hyperlipidemia, obesity, diabetes, and atrial fibrillation. The most common obstacles to effective secondary stroke prevention are motor impairment, post-stroke cognitive impairment, post-stroke depression, and stroke subtype. Long-term lifestyle modifications are difficult to sustain and require comprehensive, individualized interventions. This review underlines the benefits of adhering to lifestyle modifications as the most effective secondary stroke prevention measure.

## 1. Introduction

Stroke is a leading cause of death and disability worldwide [1], but it has been shown in several observational studies and systematic reviews to be a highly preventable disease [2,3,4]. Lifestyle modifications are essential to national/international guidelines and recommendations for secondary stroke prevention [5]. Studies have shown that 90% of strokes are caused by ten modifiable risk factors, alone or in combination [3,6]. The rate of cardiovascular mortality in people with stroke over the subsequent ten years can be reduced by up to 92% by changing lifestyle behaviors (healthy diet, physical activity, maintaining a normal body mass index, moderate alcohol consumption, and not smoking) [7]. Stroke patients often have modifiable risk factors that are unmanaged [8]. First-ever and recurrent ischemic strokes have almost the same risk factors, except for a history of previous stroke for patients with recurrent stroke [9]. In a study by Kopunek et al. that included stroke survivors in the community, 99% had one unmanaged risk factor, and 91% had two or more unmanaged risk factors [10]. People with two unmanaged risk factors have up to a three times higher risk of cardiovascular mortality [11], and multiple lifestyle risk factors increase the likelihood of poor health and all-cause mortality [10]. Furthermore, multimorbidity is concerning and is associated with poor health, significant declines, and all-cause mortality [12]. First-ever ischemic stroke is associated with an increased risk of heart disease, non-stroke vascular death, and myocardial infarction in the future [13,14,15].

Air pollution, as a new risk factor, is acknowledged as a significant contributor to stroke burden, especially in developing countries [7]. Many studies have proven the importance of primary stroke prevention interventions, whereas evidence is still lacking for secondary stroke prevention [7,9]. However, we now recognize the importance of cardiovascular risk factors for recurrent stroke [7]. Still, many widely accepted risk factors for stroke remain unattended to [8,9,10,11]. Therefore, the detection and control of risk factors are crucial in stroke patients. A prior stroke represents a critical risk for an eventual new stroke. The risk of death one month after stroke is 23% to 41%, while the rate of disability ranges from 39% to 53% [16,17,18,19]. Recurrent stroke is associated with higher mortality rates and increased disability levels [16,17,18,19,20]. One-third of strokes are recurrent, and they are 6 to 15 times more common among prior stroke sufferers than in the general population [16]. The estimated risk for a recurrent stroke is up to 16% within the first year and 4% every subsequent year, with a 5-year risk of 30% and a 10-year risk of 43% [7,16,21].

A systematic review by Lawrence M et al. found that lifestyle interventions for secondary stroke prevention can effect positive change concerning (1) lifestyle behaviors, (2) physiological outcomes, and (3) secondary outcomes. Behavioral interventions were found to be effective in increasing physical activity and improving dietary intake. Regarding physiological outcomes, interventions were effective, particularly those aiming to reduce blood pressure, cholesterol levels, and body mass index. Concerning secondary outcomes, the intervention was seen to exert a highly significant beneficial effect in improving the perceived quality of life and stroke knowledge. Behavioral changes are challenging to achieve because of complex personal, environmental, and social factors. A common barrier to behavior change is limited insight, knowledge, and awareness of how lifestyle factors contribute to the risk of secondary to subsequent stroke. If patients do not perceive a need to modify lifestyle behaviors, then behavior modification is unlikely. Even when patients understand the need to modify their behavior, the knowledge rarely translates into behavior with healthy lifestyle habits [22].

Several studies have investigated the benefits of life habit changes for secondary stroke prevention [22,23,24,25]. The most important predictors for adhering to and following recommendations [26] are high fruit and vegetable consumption [27], non-smoking [28], and exercise [29,30,31]. It was found that adopting these lifestyle modifications may lead to an 80% reduction in stroke risk [32]. Moderate-level evidence supports modifying multiple risk factors to reduce recurrent stroke [33,34,35,36], and several observational studies have shown the positive impact of lifestyle changes [7,22,25,37]. Without high-quality data, we need to be careful when interpreting these conclusions. Furthermore, educating the population has always been challenging [38]. To enable better comparisons between studies, several reports have proposed additional study protocols [39,40,41]. The key to long-term prevention is healthy habits, but in order to be successful, healthcare providers, families, and caregivers must participate in close collaboration [42,43].

A healthy lifestyle is the cornerstone of prevention. Its promotion is a major task for all healthcare providers, in collaboration with family and caregivers. It includes intervention in personal behaviors and risk factors (such as a lack of physical activity, use of tobacco, unhealthy food habits, and overweight), and all interventions aimed at changing lifestyle should be lifelong [42,43]. Importantly, engaging in healthy lifestyle behaviors protects against all-cause and cardiovascular mortality in adults with stroke [12].

In this article, we underline the benefits of adhering to lifestyle modifications as the most effective means of secondary stroke prevention.

## 2. Physical Activity

There is a consensus that stroke survivors who participate in regular physical activity may have a reduced risk of subsequent stroke [44,45]. For patients with a previous stroke, the identification and targeting of barriers to exercise delivery could lead to the more widespread implementation of exercise prescriptions in this population [45]. Lifestyle risk factors are an essential consideration among stroke survivors. These risk factors can lead to worsening symptoms and disease recurrence [45]. For successful risk reduction, communication between the physician and patient is necessary [45]. The main lifestyle steps in reducing cardiovascular mortality in stroke patients are (1) a healthy diet and regular exercise and (2) the reduction in or cessation of unhealthy habits (such as smoking and alcohol consumption) [12]. Only a tiny amount of research has assessed the effect of lifestyle changes on secondary stroke prevention. In routine practice, these necessary lifestyle changes are not made after a stroke [7]. Recurrent stroke occurs in about one-third of patients [21], and these patients are also at higher risk of other cardiovascular events [21]. Physical activity is a known preventive measure against primary and secondary stroke. The dose–effect relation is controversial, however; thus, it is not clear whether maximal physical activity also leads to maximal stroke reduction [38]. Regular physical activity has well-established benefits for reducing the risk of premature death and cardiovascular disease [46]. Physical activity has also been found to reduce the risk of coronary heart disease compared to a sedentary lifestyle. Additionally, there is no evidence that heavy physical activity confers any greater benefit than moderate levels. In recent years, accumulating evidence has supported the protective effect of moderate physical activity on stroke incidence in men and women. For stroke, the benefits are apparent even for light-to-moderate activities, such as walking, and the data support additional benefits from increasing the level and duration of one’s recreational activity [18]. Particular attention should be focused on overcoming possible barriers [45]. The further education of health professionals in this field is necessary. Exercise has proven benefits in terms of balance, walking speed, endurance, disability improvement, and metabolic function [38]. Healthy adults engage more in non-sedentary activities (57% to 72% sedentary activities) than do stroke survivors (86% to 88% sedentary) [7]. Patients who have had a stroke are particularly susceptible to sedentary and prolonged sitting behaviors. Encouraging them to engage in physical activity in a supervised and safe manner is essential. Changing their behaviors, such as diet, exercise, and medication compliance, requires more than simply providing advice or a brochure. Exercise training can be highly beneficial for people who have suffered a stroke as it can improve hypertension, lipid profiles, glucose metabolism, and insulin sensitivity. These improvements are significant, considering that up to 80% of stroke patients experience abnormal glucose metabolism. In addition to these benefits, exercise training after a stroke can improve balance, gait speed, and endurance and reduce disability [24]. The recommendations state that a sedentary lifestyle should be avoided, aside from increasing exercise [24]. However, stroke survivors often lose their exercise capacity. They need twice as much oxygen for routine walking compared to healthy individuals. The same is true with dressing, bathing, and other self-care activities; these routine activities, after stroke, take up to two-thirds of the patient’s exercise capacity. Standing and walking are good examples of non-exercise, light-intensity training. A lack of physical readiness, depressive symptoms, inaccessible environments, and physical impairment may discourage an individual from exercising, but keeping physically active has an enormous positive benefit on modifiable risk factors [7,47,48]. Exercise positively impacts the atrial fibrillation (AF) burden by reducing body weight [40,41,42,43,45]. It is still unclear whether high-intensity exercise reduces stroke occurrence, since it might increase the risk of developing AF [38]. There is increasing evidence that inactivity represents an independent AF risk factor [40,41,42]. However, it is more beneficial to influence lifestyle changes than it is to treat one single risk factor [38]. Quality of life after a stroke depends on exercise and support to perform regular training [47]. Even more significant impacts can be achieved with population-level interventions for physical activity, including investments in health-promoting infrastructure (e.g., sidewalks, walking paths, bike lanes) [23]. Additional barriers to physical activity after stroke include deconditioning, depression, inaccessible environments (e.g., wheelchair-friendly transportation and gym equipment), low motivation, poor social support, and physical impairment [23]. After an ischemic stroke or transient ischemic attack, eligible patients should exercise for half an hour (physical exercise of moderate intensity) up to five times weekly [47]. For patients with ischemic stroke or transient ischemic attack (TIA) who are capable of engaging in physical activity, at least 30 min of moderate-intensity physical exercise, typically defined as vigorous activity sufficient to break a sweat or noticeably raise heart rate, one to three times a week (e.g., walking briskly, using an exercise bicycle) may be considered to reduce the risk factors and comorbid conditions that increase the likelihood of recurrent stroke. For those individuals with a disability after ischemic stroke, supervision by a healthcare professional, such as a physical therapist or cardiac rehabilitation professional, at least on initiation of an exercise regimen, may be considered [24,47]. At a slightly higher prevalence, an estimated 57% of adults with stroke do not meet the weekly aerobic physical activity recommendations of approximately 150 min/week of moderate physical activity. This prevalence of insufficient physical activity is similar to the 62% reported among adults with disability [12]. Higher proportions of patients with TIA were noted to meet the physical activity recommendations. Insufficient levels of moderate–vigorous physical activity (typically <10 MET-hours/week) have been identified as predictive of recurrent ischemic stroke. Cardiovascular fitness is the most helpful determinant of physical activity participation and combined health behaviors after stroke, presenting a legitimate target in stroke secondary prevention. A previous systematic review also identified that cardiorespiratory fitness is highly associated with post-stroke physical activity levels. This study further showed that for every incremental increase in L/min of VO_2_peak, a stroke patient is 7.5 times more likely to meet the recommended levels of moderate–vigorous physical activity. The need for aerobic training interventions after stroke is clear, as the cardiorespiratory fitness levels of stroke patients are roughly half those of age-matched sedentary counterparts and are often insufficient to meet the threshold level required for basic activities of daily living [26]. Patients do not benefit more from intense physical activity than from moderate physical activity [18]. Further, exercise has many biological benefits related to coagulation and lipid metabolism [49]. Most stroke patients were found to have at least three modifiable unhealthy habits and two health conditions, and about one-half also had a third health condition [12]. Further, more than one-half of stroke patients did not meet weekly exercise recommendations [12]. Stroke rehabilitation starts soon after the incident and is a long-term assignment. A quick improvement in strength and mobility occurs in the first month, but the most significant improvement is reached around the fourth month. Brain remodeling continues long after this initial improvement. It is essential to remember that cognitive rehabilitation is also needed. Patients after stroke need rehabilitation regarding speech and language, dysphagia, incontinence, etc.

Aerobic exercise is essential, regardless of the patient’s specific rehabilitation needs. Enrolling patients in community exercise programs has shown particular effectiveness [50]. Recent meta-analyses suggest that office-based practices can help patients increase their levels of physical activity by 20% to 40% [51]. During the first recovery year, patients often visit their physician for concerns other than stroke. When stoke is a concern, recommended actions are often neglected [51]. Restoration of the ability to engage in physical activities stretches beyond this time; however, because of brain remodeling (i.e., healthy brain taking over the functions of the infarcted brain), the adaptation of compensating strategies, restoration of confidence, and use of adaptive equipment may be required. Effective rehabilitation therapies exist for motor recovery, cognition (i.e., memory, orientation, attention, and language), communication, incontinence, pain, dysphagia, sensory impairment (i.e., vision, neglect), spasticity, balance, and mobility. Improvements in these domains follow the same course as motor improvement, with rapid early recovery followed by a longer time to the maximum restoration of function [50]. The protective effect of physical activity may be partly mediated through its role in controlling various known risk factors for stroke. Other biological mechanisms are associated with physical activity, including reductions in plasma fibrinogen and platelet activity and elevations in plasma tissue plasminogen activator activity and high-density lipoprotein (HDL) concentrations. Thus, physical activity is a modifiable behavior that requires greater emphasis in stroke prevention campaigns [49].

## 3. Diet

Consuming a healthy diet is essential for cardiovascular health and stroke prevention. Reducing the daily caloric intake by 20% to 25% for three months or longer in obese and non-obese individuals improves blood pressure, low-density lipoprotein (LDL) cholesterol and triglycerides, insulin resistance, and glycemic control [7]. These dietary recommendations are consistent with the Dietary Approaches to Stop Hypertension (DASH) and Mediterranean-style dietary patterns, effectively reducing the risk of stroke. Adopting a Mediterranean diet, which is high in olive oil, whole grains, fruits, vegetables, and legumes and low in cholesterol/saturated fat, is advised to prevent stroke. These diets can reduce the stroke rate by 40% or more in high-risk patients [52,53]. However, supplementation with antioxidant vitamins A, C, and E or beta-carotene does not reduce the risk of stroke [54]. Several studies have indicated that a higher potassium intake reduces stroke risk by 21% (relative risk 0.79, 95% CI 0.60–0.90). This effect seems to be dose-dependent: every 1 g per day increase in potassium intake was found to reduce stroke risk by 11%. The mechanism is probably partially mediated by a reduction in blood pressure [55]. Calcium supplementation (>500 mg daily) is associated with a significant risk of myocardial infarction and a trend toward an increase in stroke [38]. Therefore, minding what we eat is a great preventive health action, and following dietary recommendations is effective in stroke reduction [7]. The Mediterranean diet has proven beneficial in stroke prevention [38], but in Western diets, salt consumption is very high at about 10 g per day. In East Europe and Asia, the amount is higher still. The amount of salt taken daily should not exceed five to six grams. Increased salt consumption raises the risk of stroke and is associated with one-fifth of all intracerebral hemorrhages. The greatest preventive impact of salt reduction comes from blood pressure reduction [38]. The consumption of all types of fat (animal, vegetable, saturated, non-saturated) does not seem to significantly influence stroke, but foods containing omega-3 fatty acids from vegetable oils decrease stroke recurrence. The consumption of industrially produced trans fats or saturated fatty acids, however, has been shown to increase the risk of coronary heart disease [56]. Protein intake, whether from animal or plant sources, may also decrease stroke recurrence. Regular consumption of fish reduces the risk of stroke, while eating meat can increase it. A diet that includes fruits and vegetables decreases the possibility of stroke recurrence. Eating only vegetables, without fruits, does not seem to be protective. A high concentration of lycopene halves the stroke risk [38].

Plant-based diets, used by vegetarians and vegans, are associated with lower body mass index and lower levels of LDL-cholesterol and blood pressure, which leads to a reduced risk of ischemic heart disease and ischemic stroke [57,58]. Interestingly, some studies show that plant-based diets might increase the risk of stroke (particularly hemorrhagic stroke), with lower levels of vitamin D, B12, calcium, and iodine [57,59]. Several meta-analyses showed that low vitamin D levels are related to an increased risk of ischemic stroke [60,61]. Moreover, low vitamin D levels are an independent factor in mortality and cardiovascular events in the general population [62]. Paradoxically, vitamin D supplementation is not associated with decreased cardiovascular events [63,64,65]. Consuming up to four cups of coffee or tea per day has a stroke-protective effect compared to no consumption, probably due to antioxidative effects and their impact on endothelial function [66]. Chocolate, with the anti-inflammatory and anti-thrombotic effects of cocoa, also has protective effects [67]. The consumption of foods and liquids with added sugars and high dietary glycemic indices increases the risk of overweight, diabetes mellitus, and coronary heart disease [68]. Dietary changes considering all the abovementioned effects may lead to a stroke risk reduction of forty percent or more in patients with high risk [69]. Family members and caregivers have a crucial role in sustaining the healthy life habits of stroke survivors [26,39]. Culture and ethnicity may play a role in this health behavior. The influence of environment, social norms, and family members on health behaviors, including fruit and vegetable intake, is well established. Studies addressing lifestyle after stroke have identified the ability of family and carers to exert both positive and negative influences on behavioral patterns, and a focus group study examining barriers to a healthy lifestyle after stroke identified that, particularly in men, the person with stroke often did not buy or prepare their own food/meals. The inclusion of family members/carers in dietary changes after stroke may be necessary to achieve sustainable behavior change, and future models of care should explore this aspect in greater depth [26]. Table 1 presents a summary of lifestyle modification recommendations.

## 4. Smoking

One of the most common habits among the population in developing countries is smoking. Smoking has a harmful effect regarding stroke; it contributes to nearly 15% of all stroke deaths yearly [70]. There is a dose-dependent relation between cigarette pack-days and stroke development. Many countries are trying to find a successful model to reduce smoking in the population. A reduction is possible through sales bans or smoking bans, but as the population increases, the total number of smokers increases yearly [52]. Nowadays, aside from cigarettes, there are several other tobacco and nicotine products available. Smoking cessation reduces the risk of developing cerebrovascular disease and stroke. The increase in the risk of stroke associated with smoking disappears four years after smoking cessation [70,71], and the risk of stroke five years after smoking cessation is the same as that in non-smokers, without any gender difference [72,73,74]. In the Multiple Risk Factor Intervention Trial, after ten years of follow-up, the risk ratio of stroke mortality associated with current smoking was 2.5, while the estimated risk ratio for stroke was 1.5 [70,71]. In the Cancer Prevention Study II, the risk of stroke mortality for current smokers versus never smokers was 1.7 in men and 2.2 in women after accounting for demographics and additional risk factors in a multivariable model [72]. The NHANES study revealed that stroke survivors who stopped smoking had lower all-cause mortality [73]; in contrast, recurrent stroke risk increases in stroke survivors who continue smoking [36].

## 5. Alcohol Consumption

Alcohol consumption is one social habit that has both a protective and a risky aspect for stroke development [75,76]. Excessive alcohol consumption and binge drinking increase the relative risk of any stroke [75]. Some studies have shown a positive effect of moderate alcohol consumption in reducing stroke and ischemic heart disease risk [75,76,77]. Moderate alcohol consumption here refers to less than two drinks daily [76]. This protective effect is usually shown as a J-shaped curve (relationship between consumption and risk of morbidity), which is also valid for dementia and diabetes type II. For other morbidities, curves might be of a linear shape (e.g., for intracerebral hemorrhage, brain atrophy, hypertension, atherosclerosis, and breast cancer) or parabolic shape (e.g., for alcoholic cirrhosis) [78]. The protective mechanism includes effects on thrombogenic blood elements and the fibrinolytic system [79]. Alcohol has the following protective effects: (1) it decreases platelet aggregation in response to adenosine-5’-diphosphate (ADP) and collagen; (2) it decreases fibrinogen, vWF, and factor VII levels; and (3) it increases the t-PA level with no change in the PAI-1 concentration (moderate consumption) [79]. However, alcohol consumption also has negative effects: (1) it inhibits platelet activation and enhances platelet activation, and (2) it decreases fibrinolysis by increasing the PAI-1 concentration (heavy consumption) [79]. Despite the protective effect on ischemic stroke, moderate alcohol consumption increases the risk for hemorrhagic stroke [76]. Chronic alcoholism also increases the risk of bleeding, mostly gastrointestinal bleeding [79]. Excessive alcohol consumption and binge drinking may lead to alcoholic cardiomyopathy and cause cardiac arrhythmias, which can precipitate thrombus formation and cause cardioembolic stroke [80]. Moreover, alcohol may trigger other diseases like obesity, atrial fibrillation, diabetes, arterial hypertension, and obstructive sleep apnea [74]. Alcohol is often packaged in the form of beverages containing a certain percentage of alcohol. The addition of sugar or other sweeteners increases the caloric load. Alcoholic liqueurs in particular contain large amounts of sugar. A high caloric intake, often a caloric surplus, may lead to obesity, diabetes, or arterial hypertension. This reduces the direct protective effect against stroke [81]. One or two glasses of wine per day for men and one glass for women may be reasonable to prevent stroke recurrence. However, strong alcoholic drinks should be avoided. Additionally, those who do not drink alcohol and who have had a stroke should not start drinking [69].

## 6. Stress

Many studies have confirmed that stress is a potential risk factor for stroke. Stress is still given insufficient consideration in everyday clinical practice [82]. The major issue is in defining stress, as it is individually dependent. A certain situation may be challenging (stressful) for one individual, while for someone else, it is a dreadful problem (causing major distress). Psychosocial stress and depression were recently confirmed as being modifiable risk factors for stroke [3]. Evaluations of long-term stress and mood disorders (e.g., depression) are necessary for patients who do not have the traditional risk factors. The same goes for chronic inflammation and subtle vascular atherosclerotic changes [83]. Several modern techniques based on mindfulness meditation have been successfully used in patients with vascular diseases, leading to the improvement in psychological problems [84]. Several studies, with a particular emphasis on reducing fatigue in some brain disorders like stroke, multiple sclerosis, and traumatic brain injury, have analyzed the potential of mindfulness-based therapies and their effectiveness in post-stroke patients [85,86]. A unique mind–body technique (TM) was found to lower blood pressure and alter some psychosocial factors. This study showed reductions in myocardial infarction, stroke, and the risk of mortality in patients suffering from coronary artery disease [87].

## 7. Air Pollution

Air pollution is a stroke risk factor that has been investigated for many years. It significantly contributes to the global burden of stroke and other cerebrovascular diseases, and it is mainly present in underdeveloped countries and in low- and middle-income countries [88]. It has short- and long-term effects on cerebrovascular and cardiovascular health. We should distinguish between indoor air (such as in households or at work) and outdoor air pollution. Indoor air pollution is much more difficult to investigate, except in the case of unique places such as factories or construction sites. Outdoor air pollution is more relevant according to the measurement and length of exposure [89]. The composition of polluted air consists of particles and several poisonous gases in low concentrations. Particles are divided by size into three groups: coarse (<10 μm size), fine (<2.5 μm size), and ultrafine (<1 μm size). The impact of air pollution on stroke also depends on the duration of exposure. The larger particles (PM_10_) are more common in industrial emissions, whereas the smaller particles (PM_2.5_ and PM_0.1_) mainly result from traffic emissions. Exposure over a short period of time refers to daily variations in different concentrations of the gases and particles in the air that constitute pollution [89]. Several studies have shown a statistically significant relationship between the duration and level of air pollution and the risk of stroke [90]. A study by Amini et al. involving 23,423 people, including 1078 stroke cases, reported a strong association between the occurrence of ischemic stroke and annual mean concentrations of coarse and fine particles in the air [91]. Another 10-year follow-up study in China described a significant occurrence of ischemic stroke after an average increase in the refined particle concentration during a mean follow-up time of 7 to 5 years [90]. A 2019 meta-analysis reported a crucial connection between an exposure period of one to four years and strokes of all types [92].

## 8. Limitations

There are several obstacles related to the relatively scarce data on secondary stroke prevention. (a) Lifestyle modification programs are not the same for all subtypes of stroke, pointing out the importance of knowing the subtype of a patient’s first-ever stroke. (b) In some patients, motor impairment following stroke precludes their active engagement in intensive physical activities. (c) Cognitive impairment, as well as post-stroke depressive changes, could also have a substantial impact. A significant obstacle in analyzing and comparing the results of different studies is the heterogeneity of definitions. The focus should be not on single changes but on multifaceted changes when considering lifestyle [93]. However, long-term lifestyle modification is difficult to sustain, and comprehensive, individualized interventions based on behavioral and medical interventions are needed. Professionals should focus on medical and lifestyle modifications to decrease the risk of stroke and other vascular diseases [93]. Long-term compliance is challenging, and many patients would rather take a pill than make changes to their lifestyle [93].

## 9. Conclusions

The evidence shows that stroke is preventable. Additionally, first and recurrent ischemic strokes have similar risk factors. Some modifiable risk factors are present in stroke patients, but they are frequently unmanaged. Despite our current knowledge of the importance and effectiveness of secondary stroke prevention, such prevention still needs greater recognition among patients, the general population, and professionals. Risk reduction on a personal level can be achieved by making lifestyle modifications: regular physical activity, a healthy diet, stress management, smoking cessation, and moderate alcohol consumption. Lifestyle modifications should address not single but multiple cardiovascular risk factors to reduce stroke risk effectively. Long-term life habit modification is not easy to sustain; interventions should be comprehensive and individualized.

## Figures and Tables

**Table 1:** Summary of recommendations regarding personal lifestyle modifications.

Lifestyle Modification	Recommendation
Physical activity	150 min/week of moderate physical activity
Healthy diet	DASH diet * or Mediterranean diet
Smoking	Smoking cessation
Alcohol consumption	Moderate (up to 2 drinks/day)
Stress	Relaxation techniques Treatment of mood disorders (depression)

* Dietary Approaches to Stop Hypertension (DASH).
